# An Electrocardiographic System With Anthropometrics via Machine Learning to Screen Left Ventricular Hypertrophy among Young Adults

**DOI:** 10.1109/JTEHM.2020.2990073

**Published:** 2020-04-24

**Authors:** Gen-Min Lin, Kiang Liu

**Affiliations:** 1 Department of Preventive MedicineNorthwestern University Feinberg School of Medicine 12244 ChicagoIL60611USA; 2 Department of MedicineHualien Armed Forces General Hospital 63426 Hualien97144Taiwan; 3 Tri-Service General HospitalNational Defense Medical Center 71548 Taipei11490Taiwan; 4 Department of Preventive MedicineNorthwestern University Feinberg School of Medicine 12244 ChicagoIL60611USA

**Keywords:** Anthropometrics, electrocardiographic system, left ventricular hypertrophy, machine learning, young adults

## Abstract

The prevalence of physiological and pathological left ventricular hypertrophy (LVH) among young adults is about 5%. A use of electrocardiographic (ECG) voltage criteria and machine learning for the ECG parameters to identify the presence of LVH is estimated only 20-30% in the general population. The aim of this study is to develop an ECG system with anthropometric data using machine learning to increase the accuracy and sensitivity for a screen of LVH. In a large sample of 2,196 males, aged 17–45 years, the support vector machine (SVM) classifier is used as the machine learning method for 31 characteristics including age, body height and body weight in addition to 28 ECG parameters such as axes, intervals and voltages to link the output of LVH. The diagnosis of LVH is based on the echocardiographic criteria for young males to be 116 gram/meter^2^ (left ventricular mass (LVM)/body surface area) or 49 gram/meter^2.7^ (LVM/body height^2.7^). On the purpose of increasing sensitivity, the specificity is adjusted around 70-75% and all data tested in proposed model reveal high sensitivity to 86.7%. The area under curve (AUC) of the Precision-Recall (PR) curve is 0.308 in the proposed model which is better than 0.109 and 0.077 using Cornell and Sokolow-Lyon voltage criteria for LVH, respectively. Our system provides a novel screening tool using age, body height, body weight and ECG data to identify most of the LVH among young adults. It provides a fast, accurate and practical diagnosis tool to identify LVH.

## Graphical abstract

The aim of this study was to develop an ECG system with anthropometric data using machine learning to increase the accuracy and sensitivity for a screen for physiological and pathological left ventricular hypertrophy (LVH) among young adults. In a large sample of 2,196 males aged 17-45 years, the support vector machine (SVM) classifier was used as the machine learning method for 31 characteristics, including age, body height and body weight, in addition to 28 ECG parameters such as axes, intervals and voltages to link the output of LVH. Our system provides a novel screening tool using age, body height, body weight and ECG data to identify most of the LVH among young adults. It provides a fast, accurate and practical diagnosis tool to identify LVH.

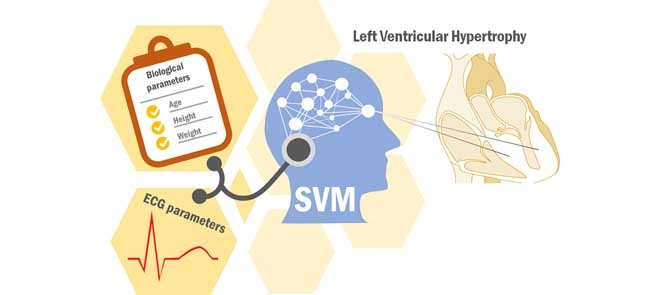

## I. Introduction

Artificial intelligence (AI) grows fast with the improvement of technology and the availability of various kinds of big data. Machine learning, an AI of the computational statistics, has been introduced in clinical medicine which could provide accurate diagnosis of disease and prediction of the risk [1]–[12]. For example, [12] utilizes the random survival forest technique identifying the top-20 risk factors of cardiovascular events and the performance is superior to the traditional risk calculators. In the modern era, using machine learning techniques has become an efficient and reliable tool for clinical practice by physicians globally.

Left ventricular hypertrophy (LVH), which is clinically considered as a sign of end-organ damage related to long-term hypertension, has been associated with heart failure and cardiovascular disease events among middle and old-aged individuals [13], [14]. In contrast, the prevalence of LVH in young adults is low, accounting for approximately 5% [15], and the phenotypes are usually caused by physiologic adaptions to intense physical training [16] and congenital hypertrophic cardiomyopathy [17]. A prior population research also shows that the presence of LVH at young ages is associated with higher risk of incident cardiovascular disease events [18]. The 12-lead surface electrocardiography (ECG) is the currently most used tool for screening the presence of LVH in the general population [19]. Several ECG-based criteria such as the Cornell and Sokolow-Lyon formulas have been proposed for more than 30 years [20], [21]; however, the performance of the ECG-based criteria for LVH consistently yields high specificity (>95%) but low sensitivity (20%-30%). Over the past 5 years, a few population studies were presented by machine learning and deep learning for the ECG characteristics to detect presence of LVH [1]–[3]. For hypertrophic cardiomyopathy (HCM), one of the most common pathological LVH, Rahman *et al.* firstly used the random forest and the support vector machine (SVM) techniques, and 5-fold cross validations, for hundreds of ECG characteristics training, where showed excellent results regarding the sensitivity, specificity and precision up to 90% in a hospital-based population study [2]. Subsequently, Tison *et al.* used the deep learning of convolutional neural network for numerous ECG parameters to identify HCM, consistently showing excellent results in a hospital-based population study [3]. However, the sensitivity using the specific ECG criteria for HCM has approached up to 90% [22], [23]. By contrast, a community-based population study using machine learning for the ECG to screen any unspecific LVH was proposed by Sparapani *et al.* [1]. Despite this study utilized the tree-based, Bayesian nonparametric machine learning technique for a number of ECG characteristics alone, the sensitivity for detecting any unspecific LVH phenotype in a general population of middle and old aged individuals is increased up to 29.0%, merely a little improvement compared with the other ECG criteria for LVH [1]. Accordingly, computerized training of the ECG alone might not be adequate in screening for unspecific LVH in the general population level.

**FIGURE 1.**
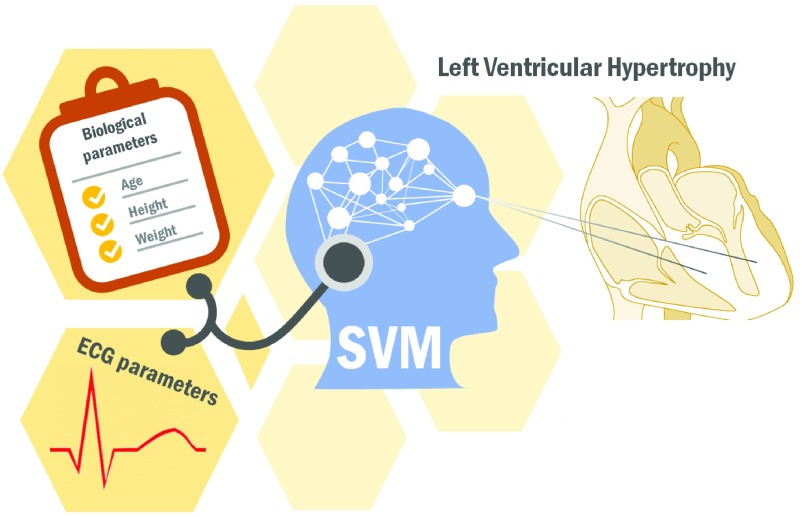
Schematic diagram of proposed system.

In this paper, we aim to develop a clinically accessible ECG-based system which uses a large sample of the military young personnel taking age, anthropometric data and several ECG characteristics into account for machine learning by the method of SVM to predict the presence of unspecific LVH as shown in Fig. 1. The rest of this paper is organized as follows. The materials are presented in Section II. In Section III, the proposed algorithm regarding the ECG system for unspecific LVH detection is described in detail. Section IV displays the experimental results. Section V concludes this paper.

## II. Data Collection and Features Selection

### A. Data Collection

This study uses a population of 2,196 military males aged 17–45 years from the ancillary cardiorespiratory fitness and hospitalization events in armed forces (CHIEF) substudy performed in the Hualien Armed Forces General Hospital in Hualien city, Taiwan, R.O.C. Each participant received an ECG and an echocardiography at the same clinic visit in a health examination prior to the annual exercise tests for the military rank promotions and awards. The study design has been described in detail previously [24]–[33]. The raw data of 12-lead ECG parameters are interpreted by the software products of two ECG manufacturers: one is the CARDIOVIT MS-2015 (Schiller AG, Baar, Switzerland), and another one is the TC70 CARDIOGRAPH (Philips, Amsterdam, Netherlands). The transthoracic echocardiography is performed via the IE33 (Philips, Amsterdam, Netherlands). All the echocardiography and ECG procedures are implemented by the same technician who has been certificated with plenty of experiences for longer than 20 years. The 28 ECG characteristics adopted in the proposed method include heart rate, the durations of P wave, PR interval, QRS interval, QT interval and QTc interval in Lead II, and the axes of P, QRS, and T waves in Lead II, and the voltages of R waves in all Limb Leads I, II, III, aVR, aVL, aVF and S wave in Lead aVL, and the voltages of R and S waves in all precordial Leads V1-V6, where the voltage of 1 mV indicates 10 mm. In addition, a population of 203 military females aged 17–42 from the ancillary CHIEF substudy is utilized as an additional test set using the male model of machine learning by age, anthropometric data and ECG parameters. The comparison methods are the Sokolow-Lyon voltage criterion for LVH [20] and the Cornell voltage criteria for males and females [21], which are revealed in Table 1, respectively.

**TABLE 1:** Electrocardiographic and Echocardiographic Criteria for Left Ventricular Hypertrophy

	Criteria	Sex-specific	Description
Electrocardiographic	Sokolow-Lyon voltage	Both Sexes	(S-V1 or S-V2+ R-V5 or R-V6) ≥ 35 mm
	Cornell voltage	Male Adults	R-aVL+S-V3 ≥ 28 mm
		Female Adults	R-aVL+S-V3 ≥ 20 mm
Echocardiographic	CARDIA-based	Young Male Adults	LVM/BSA ≥ 116 gram/meter^2^ or LVM/height^2.7^≥ 49 gram/meter^2.7^
	CHIEF-based	Young Female Adults	LVM/BSA ≥ 88 gram/meter^2^ or LVM/ height^2.7^ ≥ 41 gram/meter^2.7^

Abbreviations: CARDIA, coronary artery risk development in young adults; CHIEF, cardiorespiratory fitness and hospitalization events in armed forces

The diagnosis of LVH is based on the recommendations of the American Society of Echocardiography [34]. Quantification of left ventricular internal dimension (LVIDd) and left ventricular wall thickness including interventricular septum (IVSd) and left ventricular posterior wall (LVPWd) is measured by M-mode and 2-dimensional methods at the mitral valve tips and at the onset of the QRS complex in ECG of end diastole in echocardiographic parasternal long axis view. Left ventricular mass (LVM) is calculated on the basis of the corrected echocardiographic formula proposed by Devereux *et al.* [35] as shown in (1).

\begin{align*}&\hspace {-2pc} \mathrm {LVM=0.8\times [1.04\times }{\mathrm {(LVIDd+IVSd+LVPWd)}}^{3} \\&\qquad \qquad \qquad \qquad \qquad \qquad \quad -{\mathrm {LVIDd}}^{3}]+0.6\tag{1}\end{align*}

LVM is respectively indexed for body surface area (BSA) and for height$^{2.7}$ based on the Dubois and Dubois [36] and de Simone *et al*. formula [37], respectively. Echocardiographic LVH for young males is defined to be the $95^{\mathrm {th}}$ percentile of the military males and according to the finding of a prior Coronary Artery Risk Development in Young Adults study (CARDIA) [18]. In addition, echocardiographic LVH for young females is defined according to the $95^{\mathrm {th}}$ percentile of the military females in the CHIEF study and the results of a prior study for Southeastern Asia young females [38]. The sex-specific echocardiographic criteria for LVH are listed in Table 1. To develop the proposed machine learning method, the data are partitioned into 80% for cross validation and 20% for test for the male samples. The study protocol was approved by the Institutional Review Broad of Mennonite Christian Hospital (No. 16-05-008) in Hualien City, Taiwan.

### B. Pre-Test for Input Features

To select the proper features, at initial stage, we stepwise add several biological parameters on the 28 ECG parameters, as input features for SVM machine learning to determine the most clinically efficient system. These biological parameters include age, body height, body weight, body mass index (BMI), waist circumference, systolic blood pressure (SBP), diastolic blood pressure (DBP) and body surface area (BSA). The preliminary performances of additional biological parameters and adopted 28 ECG parameters are listed in Table 2. For the stepwise pre-test of input features, we only take training set and test set without cross validation for SVM model to compare the results of various ECG-based combinations. As shown in Table 2, when more input parameters are trained, there are larger area under curves (AUCs) of the Receiver Operating Characteristic (ROC) curves and Precision-Recall (PR) curves in the test set. A significant improvement in AUCs of the ROC and PR curves is observed when using age, body height, body weight with the 28 ECG parameters as inputs to relate to the output of LVH. Additional inputs of BMI, waist circumference, BSA, SBP and DBP are neutral or merely increase a little in performance. Thus, the 31 features including age, body height, body weight, and the 28 ECG parameters are determined as the input features of our machine learning model. The average values in each parameter of the participants are revealed in Table 3. The label of LVH is by the echocardiographic LVM/BSA ≥ 116 gram/meter^2^ or LVM/height^2.7^ ≥ 49 gram/meter^2.7^ for young males. As shown in Table 3, the characteristics in those with and those without LVH are continuous data which are expressed as mean ± standard deviation and compared by two samples t-test. A p-value < 0.05 is considered significant. Notably, older age, lower body height and greater body weight are observed in those with echocardiographic LVH.

**TABLE 2:** Preliminary Performance of Additional Biological Parameters and Adopted 28 ECG Parameters

ECG (28)	✔	✔	✔	✔	✔	✔	✔
Age	–	✔	✔	✔	✔	✔	✔
Height and Weight	–	–	✔	✔	✔	✔	✔
BMI	–	–	–	✔	✔	✔	✔
Waist Circumference	–	–	–	–	✔	–	✔
SBP and DBP	–	–	–	–	–	✔	✔
BSA	–	–	–	–	–	–	✔
Number of input features	28	29	31	32	33	34	36
ROC AUC	0.765	0.772	0.855	0.858	0.860	0.857	0.866
PR AUC	0.215	0.214	0.303	0.311	0.314	0.322	0.339

**TABLE 3:** Characteristics of Study Participants (Males)

Features	Total N=2196	Non-LVH N=2046	LVH N=150	p-value
Age (years)	25.93± 6.77	25.68± 6.67	29.41± 7.18	< 0.001
Height (cm)	172.01± 5.92	172.19± 5.89	169.55± 5.68	< 0.001
Weight (kg)	72.47± 12.03	71.86± 11.73	80.73± 13.06	< 0.001
Heart rate (bpm)	67.38± 11.83	67.47± 11.69	66.03± 13.53	0.206
P-II(ms)	105.85± 15.26	105.80± 15.17	106.57± 16.49	0.551
PR-II(ms)	157.91± 21.02	157.40± 20.97	164.91± 20.45	< 0.001
QRS-II(ms)	97.26± 10.75	97.11± 10.70	99.28± 11.24	0.017
QT-II(ms)	372.21± 29.64	371.21± 29.28	385.82± 31.24	< 0.001
QTc-II(ms)	390.14± 24.56	389.71± 24.30	396.01± 27.34	0.007
P axis-II(degree)	44.78± 27.21	45.41± 27.20	36.09± 25.99	< 0.001
QRS axis-II(degree)	65.22± 32.23	65.92± 31.96	55.71± 34.34	< 0.001
T axis-II(degree)	35.87± 21.37	36.52± 21.14	27.03± 22.65	< 0.001
R-I(mm)	5.81± 3.00	5.65± 2.90	7.99± 3.43	< 0.001
R-II(mm)	12.86± 4.93	12.97± 4.91	11.35± 5.02	< 0.001
R-III(mm)	8.53± 5.83	8.71± 5.86	6.04± 4.81	< 0.001
R-aVR(mm)	1.62± 2.31	1.58± 2.26	2.14± 2.79	0.019
R-aVL(mm)	2.34± 2.23	2.24± 2.10	3.73± 3.24	< 0.001
S-aVL(mm)	2.21± 2.98	2.28± 3.02	1.31± 2.23	< 0.001
R-aVF(mm)	10.50± 5.34	10.67± 5.33	8.20± 4.80	< 0.001
R-V1(mm)	3.54± 2.28	3.55± 2.27	3.37± 2.45	0.347
S-V1(mm)	10.25± 5.30	10.27± 5.25	9.96± 6.01	0.542
R-V2(mm)	8.58± 4.24	8.55± 4.19	9.08± 4.90	0.199
S-V2(mm)	16.01± 6.89	16.08± 6.92	15.04± 6.52	0.075
R-V3(mm)	13.26± 6.23	13.20± 6.22	14.02± 6.26	0.119
S-V3(mm)	8.45± 5.34	8.39± 5.30	9.29± 5.80	0.045
R-V4(mm)	19.73± 6.84	19.80± 6.87	18.78± 6.44	0.076
S-V4(mm)	5.15± 4.10	5.10± 4.09	5.82± 4.28	0.039
R-V5(mm)	19.86± 5.86	19.85± 5.83	19.95± 6.31	0.848
S-V5(mm)	3.17± 2.93	3.14± 2.91	3.49± 3.18	0.168
R-V6(mm)	16.35± 5.08	16.32± 5.06	16.84± 5.32	0.223
S-V6(mm)	1.86± 1.99	1.85± 1.98	1.92± 2.20	0.721

## III. Proposed Method

**FIGURE 2.**
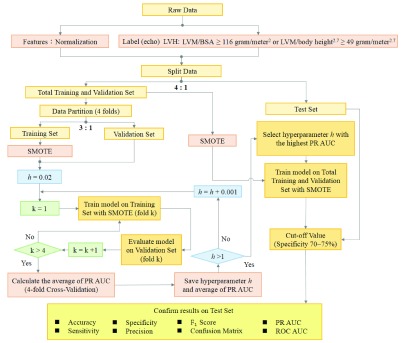
Flowchart of the proposed method.

According to the preliminary pre-test outcomes, the 31 input factors for machine learning are age, body height, body weight and the adopted 28 ECG parameters. This paper uses the SVM for predicting the presence of LVH among young adults. The reason for selecting SVM as the model is due to the advantages of SVM classifier which are effective in high dimensional spaces and memory efficient, and could provide successful discriminative models in many fields [2], [39]–[41]. In addition, the training time and running time of SVM are extremely short. Therefore, we utilize the SVM machine learning technique which can be feasible in an ECG equipment to achieve practical application. The flowchart of the proposed method is illustrated in Fig.2.

### A. Data Normalization

Firstly, we use the normalization of Min-Max scaling [42], [43] to individually normalize the original data of 31 input features into the interval between 0~1 for solving the problem of different dynamic ranges of various input features. Min-Max normalization performs a linear transformation on the original data. Each of the actual data $d$ of feature $f$ is mapped to a normalized value which lies in the range of 0 to 1. The Min-Max normalization is calculated by using (2).

\begin{equation*} Normalized\left ({d }\right)=d'=\frac {d-min(f)}{max(f)-min(f)}\tag{2}\end{equation*}

where $d$ indicates the original data of feature $f$ among the 31 input features, min($f$) and max($f$) represent the minimum and maximum values of the input feature $f$, respectively. $d'$ denotes the normalized data.

### B. Cross Validation

The data of 2,196 military males are segmented into a total training and validation set and a test set with 4:1 ratio. The total training and validation set is partitioned into four equal size groups. Among the four groups, one group is treated as the validation set for validating the model, and the remaining three groups are taken as the training set. Each of the four groups is used once as the validation set. The proportions of non-LVH and LVH cases are similar across each group. The cross validation process is then repeated four times. Four AUCs of PR curves from the four folds are averaged as a single performance of the results. By using a 4-fold cross validation, a better generalization assessment of the performance for training can be obtained.

### C. Application of Smote

The data numbers illustrated by four folds are described in detail in Table 4. Our datasets are predominately composed of non-LVH cases with only a small percentage of LVH cases since the prevalence of LVH in young adults is approximately 5%. For example, in Table 4, the 1st cross validation, the numbers of the training set and validation set are 1,317 (Non-LVH: 1,225, LVH: 92) and 439 (Non-LVH: 408, LVH: 31), respectively. This imbalance in sample sizes between the Non-LVH and LVH cases is obvious. The solution for this issue is to increase LVH cases in pre-processing by applying the synthetic minority over-sampling technique (SMOTE) [44]. The main idea of SMOTE is to create new minority class samples by choosing a near minority class neighbor randomly and interpolating as described as follows. Firstly, for each minority class sample $S_{i}$, its $k$ nearest neighbors from other minority class samples are taken. Secondly, minority class sample $S_{j}$ among the $k$ neighbors is randomly selected. Finally, the $S_{New}$ is generated as the synthetic sample by interpolating between $S_{i}$ and $S_{j}$ as (3).

\begin{equation*} S_{New}=S_{i}+rand\left ({0,1 }\right)\ast \left ({S_{j}-S_{i} }\right)\tag{3}\end{equation*}

where *rand* (0, 1) stands for a random number between 0 and $1.~S_{j} \in ${$k$ neighbors of $S_{i}$}. The process of applying SMOTE can be treated as interpolating between two LVH samples in the viewpoint of geometry. The decision space for the LVH samples is expanded. Thus, it allows the SVM method to have a higher prediction performance on unknown LVH samples. The SMOTE is used in the process of 4-fold cross validation. The training data of the LVH group are pre-processed and augmented by SMOTE to be the same numbers with those of the Non-LVH group as 1,225, 1,230, 1,229 and 1,215, respectively, for the four folds as shown in Table 4. We also compare the performances of the test set for the proposed model with and without using SMOTE.

**TABLE 4:** Data Numbers in the Training and Validation Set

Fold	Data	Non-LVH	LVH	Total
1st	Training Set	1225	92	1317
	Pre-processed by SMOTE	1225	1225	2450
	Validation Set	408	31	439
2nd	Training Set	1230	87	1317
	Pre-processed by SMOTE	1230	1230	2460
	Validation Set	403	36	439
3rd	Training Set	1229	88	1317
	Pre-processed by SMOTE	1229	1229	2458
	Validation Set	404	35	439
4th	Training Set	1215	102	1317
	Pre-processed by SMOTE	1215	1215	2430
	Validation Set	418	21	439

### D. Machine Learning Model

Our proposed method utilizes SVM [45]–[48] as a binary classifier for machine learning. In our method, a 31-dimensional vector represents a data point and we employ the linear kernel (Linear SVM) to separate such points by a 30-dimesional hyperplane. Maximum-margin is constructed by SVM so that the distance from the hyperplane to the nearest subset of the training data points (support vectors) of Non-LVH or LVH class is maximized. The soft-margin SVM is adopted in our method. Soft-margin SVM allows the wide decision margin and some outliers are inside or on the wrong side of the margin.

Let $x_{i} \in ~\text{R}^{31}$ denote a 31-dimensional training vector with associated label $y_{i} \in $ {1, −1}. $x_{i}$ also includes the synthetic samples of LVH group applied by SMOTE and all the data of $x_{i}$ are processed by Min-Max normalization. $n$ indicates the number of training vectors. The weight vector $w$, which is related to the construction of hyperplane for SVM, is obtained by solving the objective function as shown in (4) [41], [47]. The second term in (4) is the squared hinge loss (L2 loss) function for the soft-margin SVM evaluated on the training data and weighted by hyperparameter $h$. The soft-margin formulation can help in avoiding over-fitting.

\begin{equation*} {\min \limits _{w}}~\,{\frac {1}{2}w^{T}w+h}\sum \limits _{i=1}^{n} {{(\mathrm {max}(0,1-y_{i}w^{T}x_{i}))}^{2}}\tag{4}\end{equation*}

where $h$ is a hyperparameter which decides the trade-off between maximizing the margin and minimizing the training error. When $h$ is large, avoiding misclassification is emphasized at the expense of maintaining the margin small, whereas when $h$ is small, classification errors are presented less importance and focus is more on maximizing the margin. The optimized hyperparameter $h$ is chosen by grid search according to the average AUC of the PR curves of the cross validation in our algorithm. As demonstrated in Fig.2, the hyperparameter $h$ is initialized to 0.02. The training processes with the increment 0.001 of $h$ for grid search is iterated until $h$ reaches to 1. The optimized hyperparameter is chosen based on the highest AUC of the PR curves among the candidates of $h$.

After selecting the optimized hyperparameter, the SVM training model will be determined by the data in the total training and validation set. As shown in Table 5, the data of total training and validation set for the LVH group are pre-processed by SMOTE, and the number is increased to 1,633.

**TABLE 5:** Data Numbers of Total Data

Data	Non-LVH	LVH	Total
Total Training and Validation Set	1633	123	1756
Pre-processed by SMOTE	1633	1633	3266
Test Set	413	27	440
Total Data	2046	150	2196

## IV. Results and Discussion

Our proposed method is implemented using the software scikit learn v0.20.2 with Python programming language [49]. In addition, the optimal weight vector $w$ for hyperplane is obtained by LIBLINEAR (A Library for Large Linear Classification) [47], an open source library for large linear classification. The optimized hyperparameter $h~0.322$ is chosen when the highest AUC of the PR curve averaged from the 4-fold cross validation is found from the values of 981 trials.

### A. Performance Measurement

The specificity 70-75% is the criterion to decide the most appropriate test cut-off probability [50] for our SVM method. The performance is assessed by several standard measurements including accuracy, specificity, sensitivity (recall), precision, F_1_ score, the AUC of the ROC curve and the AUC of the PR curve [51], [52].

The definitions of accuracy, specificity, sensitivity and precision are calculated by true positive (TP), true negative (TN), false positive (FP), and false negative (FN) as denoted in (5) - (8). The F_1_ score, which is the harmonic average of the precision and recall, is described in (9).

\begin{align*} Accuracy=&\frac {TP+TN}{TP+TN+FP+FN} \tag{5}\\ Specificity=&\frac {TN}{TN+FP} \tag{6}\\ Sensitivity(Recall)=&\frac {TP}{TP+FN} \tag{7}\\ Precision=&\frac {TP}{TP+FP} \tag{8}\\ F_{1}~score=&\frac {2\times Precision\times Recall}{Precision+Recall}\tag{9}\end{align*}

### B. Results

**FIGURE 3.**
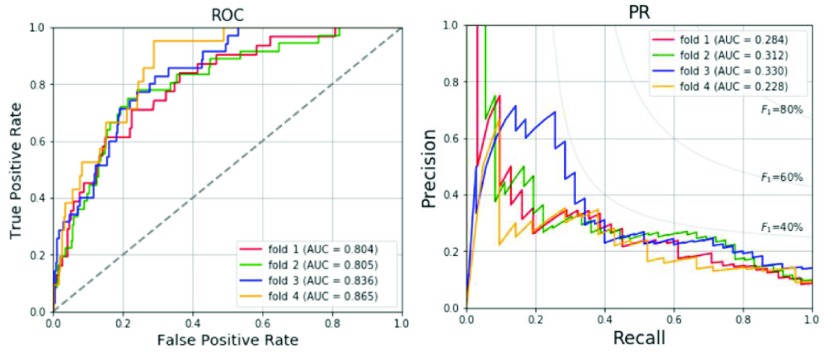
ROC and PR curves for the 4-fold cross validation.

**FIGURE 4.**
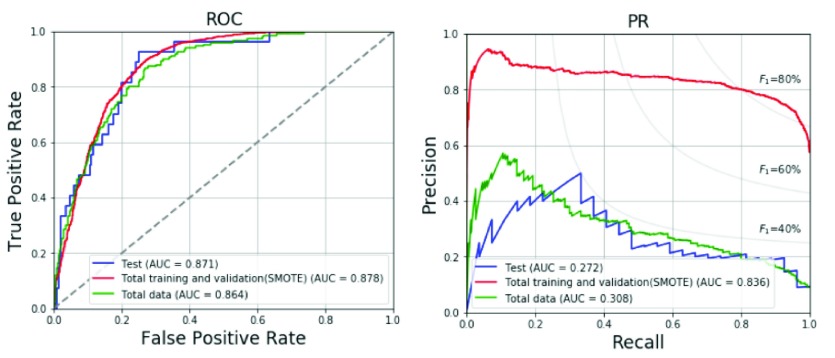
ROC and PR curves of the proposed method for various datasets.

The results of the 4-fold cross validation for the validation set with the optimized hyperparameter are shown in Table 6. The prevalence of LVH in the validation set is range from 4.8% to 8.2% as shown in Table 6. Average accuracy, specificity, sensitivity, precision and F_1_ score are 73.3%, 72.9%, 78.8%, 18.3% and 29.3%, respectively. The ROC and PR curves for the four folds are compared in Fig. 3. The average AUC of the ROC curve is 0.828 and the average AUC of the PR curve is 0.289. Table 7 shows the prediction results of the total training and validation set, test set and total data. In the total training and validation set, the SMOTE is applied for the LVH group to increase the prevalence rate to 50%. Thus, the precision, F_1_ score and AUC of PR curves are much better than the other two datasets. In the test set and total data, the prevalence of LVH is generally distributed around 6-7% in the population of young adults. The results of the test set for the model with SMOTE regarding the accuracy, specificity, sensitivity, precision and F_1_ score are 76.1%, 75.1%, 92.6%, 19.5% and 32.2%, respectively, which are in line with the results of the total data for the model with SMOTE, and better than 74.6%, 73.9%, 85.2%, 17.6%, and 29.2%, respectively, for the model without using SMOTE. The ROC and PR curves for various datasets are compared in Fig. 4. The AUC values for the three datasets are similar in ROC curves. The AUC values of ROC and PR curves of the proposed method with SMOTE for test set are 0.871 and 0.272, respectively, and larger than 0.841 and 0.259, respectively, for the model without using SMOTE.

**TABLE 6:** Data Numbers and Performances for the 4-Fold Cross Validation

	Validation set 1st fold	Validation set 2nd fold	Validation set 3rd fold	Validation set 4th fold	Average
Non-LVH Group	408	403	404	418	–
LVH Group	31	36	35	21	–
Total	439	439	439	439	–
Prevalence Rate	7.1%	8.2%	8.0%	4.8%	–
Accuracy	67.2%	77.9%	75.9%	72.0%	73.3%
Specificity	66.4%	78.2%	75.7%	71.3%	72.9%
Sensitivity	77.4%	75.0%	77.1%	85.7%	78.8%
Precision	14.9%	23.5%	21.6%	13.0%	18.3%
F_1_-score	25.0%	35.8%	33.7%	22.6%	29.3%
ROC AUC	0.804	0.805	0.836	0.865	0.828
PR AUC	0.284	0.312	0.330	0.228	0.289
True Negative	271	315	306	298	–
False Negative	7	9	8	3	–
False Positive	137	88	98	120	–
True Positive	24	27	27	18	–

**TABLE 7:** Predicted Results of the Proposed Method for Various Datasets

	Total Training and validation set (SMOTE)	Test set	Total data
Non-LVH Group	1633	413	2046
LVH Group	1633	27	150
Total	3266	440	2196
Prevalence Rate	50.0%	6.1%	6.8%
Accuracy	81.2%	76.1%	74.2%
Specificity	72.8%	75.1%	73.3%
Sensitivity	89.6%	92.6%	86.7%
Precision	76.7%	19.5%	19.2%
F_1_-score	82.6%	32.2%	31.4%
ROC AUC	0.878	0.871	0.864
PR AUC	0.836	0.272	0.308
True Negative	1189	310	1499
False Negative	170	2	20
False Positive	444	103	547
True Positive	1463	25	130

**FIGURE 5.**
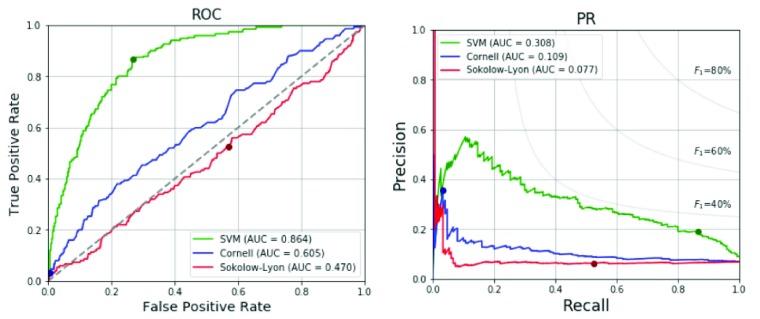
ROC and PR curves of the proposed method and traditional ECG voltage criteria.

Our proposed SVM machine learning method is also compared with the Sokolow-Lyon voltage and Cornell voltage criteria for LVH as shown in Table 8. All data of the 2,196 military males are tested in the model. With the specificity of 73.3%, intended to be set between 70-75%, our SVM technique provides much better sensitivity 86.7% compared to 3.3% and 52.7% regarding the Cornell and Sokolow-Lyon voltage criteria, respectively. The ROC and PR curves for the three approaches are shown in Fig. 5. It is obvious that the proposed method is much superior to the other two traditional ECG voltage criteria.

**TABLE 8:** Performance Comparison of Proposed Method and Traditional ECG Voltage Criteria

	SVM	Cornell voltage	Sokolow-Lyon voltage
Cut-off Value	0.422	28 mm	35 mm
Accuracy	74.2%	93.0%	43.8%
Specificity	73.3%	99.6%	43.2%
Sensitivity	86.7%	3.3%	52.7%
Precision	19.2%	35.7%	6.4%
F_1_-score	31.4%	6.0%	11.4%
ROC AUC	0.864	0.605	0.470
PR AUC	0.308	0.109	0.077
True Negative	1499	2037	883
False Negative	20	145	71
False Positive	547	9	1163
True Positive	130	5	79

**FIGURE 6.**
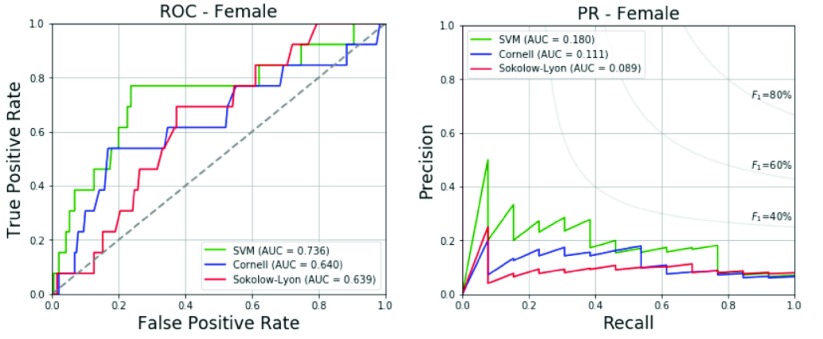
ROC and PR curves of the proposed method and traditional ECG voltage criteria for the female’s test data.

In addition, we also test the CHIEF military female subcohort data with the label of echocardiographic LVH by the definitions of LVM/BSA ≥ 88 gram/meter^2^ or LVM/height^2.7^ ≥ 41 gram/meter^2.7^ for young females using the proposed SVM model trained by the military young males. The baseline data with an average for each adopted biological and ECG features of the female participants with and without echocardiographic LVH are revealed in Table 9. It is contrary to the findings of the male participants that there are no significant differences in the adopted biological parameters including age, body height and body weight, and there are only two significant differences in the ECG characteristics including the amplitudes of R waves in chest Leads V1 and V3. In addition, the results of the female test set with regard to the accuracy, specificity, sensitivity, precision and F_1_ score are 76.4%, 76.3%, 76.9%, 18.2% and 29.4%, respectively, and shown in detail in Table 10. Compared to the conventional Sokolow-Lyon voltage and Cornell voltage criteria specifically for females [21], the proposed SVM method can provide superior performance evaluated by F_1_ score, and the AUCs of the ROC and PR curves. The ROC and PR curves obtained from the female’s test data are shown in Fig. 6.

**TABLE 9:** Characteristics of Study Participants (Females)

Features	Total N=203	Non-LVH N=190	LVH N=13	p-value
Age (years)	25.42± 5.38	25.47± 5.31	24.69± 6.56	0.6162
Height (cm)	160.55± 4.81	160.63± 4.75	159.35± 5.72	0.3570
Weight (kg)	58.90± 9.24	58.55± 8.83	64.00± 13.34	0.1711
Heart rate (bpm)	68.40± 11.72	68.23± 11.24	71.00± 17.77	0.5884
P-II(ms)	100.07± 15.99	99.90± 15.60	102.54± 21.48	0.5661
PR-II(ms)	148.78± 23.52	149.41± 23.63	139.62± 20.53	0.1470
QRS-II(ms)	89.93± 12.10	89.55± 11.23	95.46± 21.00	0.3338
QT-II(ms)	383.96± 34.32	383.16± 32.74	395.54± 52.76	0.4193
QTc-II(ms)	410.01± 29.14	408.98± 27.24	425.08± 48.41	0.2576
P axis-II(degree)	42.16± 28.33	42.83± 28.22	32.31± 29.16	0.1957
QRS axis-II(degree)	71.02± 31.76	71.65± 30.85	61.92± 43.47	0.2866
T axis-II(degree)	33.94± 20.82	34.18± 20.06	30.31± 30.80	0.6621
R-I(mm)	4.19± 2.16	4.11± 2.08	5.36± 3.02	0.1647
R-II(mm)	11.38± 3.75	11.32± 3.70	12.22± 4.48	0.4014
R-III(mm)	8.34± 4.61	8.36± 4.57	8.07± 5.37	0.8273
R-aVR(mm)	0.90± 1.17	0.93± 1.18	0.57± 0.83	0.2879
R-aVL(mm)	1.46± 1.18	1.42± 1.15	1.93± 1.47	0.1330
S-aVL(mm)	2.69± 2.80	2.72± 2.81	2.38± 2.70	0.6813
R-aVF(mm)	9.83± 3.92	9.81± 3.92	10.12± 4.03	0.7816
R-V1(mm)	2.75± 1.60	2.67± 1.53	3.92± 2.10	0.0059
S-V1(mm)	7.03± 3.52	6.91± 3.51	8.72± 3.37	0.0732
R-V2(mm)	6.42± 2.88	6.34± 2.80	7.70± 3.70	0.0983
S-V2(mm)	9.78± 4.82	9.70± 4.76	11.02± 5.76	0.3387
R-V3(mm)	9.20± 4.40	9.03± 4.30	11.68± 5.14	0.0348
S-V3(mm)	5.50± 3.76	5.41± 3.68	6.83± 4.79	0.1872
R-V4(mm)	13.24± 4.45	13.16± 4.42	14.38± 4.91	0.3411
S-V4(mm)	3.64± 2.94	3.61± 2.91	4.21± 3.46	0.4768
R-V5(mm)	13.52± 3.96	13.45± 3.98	14.45± 3.55	0.3824
S-V5(mm)	2.53± 2.19	2.51± 2.16	2.70± 2.60	0.7664
R-V6(mm)	12.09± 3.80	12.01± 3.78	13.15± 4.08	0.2956
S-V6(mm)	1.61± 1.57	1.62± 1.55	1.54± 1.87	0.8512

**TABLE 10:** Performance Comparison of Proposed Method and Traditional ECG Voltage Criteria for Female’s Test Data

	SVM	Cornell voltage	Sokolow-Lyon voltage
Cut-off Value	0.355	20 mm	35 mm
Accuracy	76.4%	93.1%	91.6%
Specificity	76.3%	99.5%	97.4%
Sensitivity	76.9%	0%	7.7%
Precision	18.2%	0%	16.7%
F_1_-score	29.4%	0%	10.5%
ROC AUC	0.736	0.640	0.639
PR AUC	0.180	0.111	0.089
True Negative	145	189	185
False Negative	3	13	12
False Positive	45	1	5
True Positive	10	0	1

**FIGURE 7.**
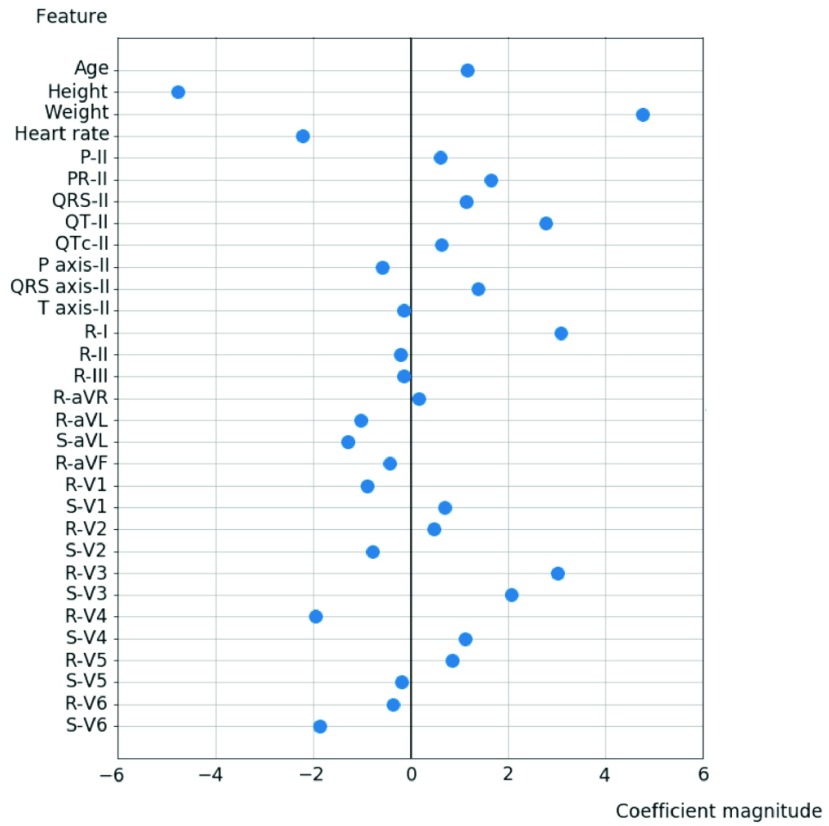
Feature importance of the 31 input parameters.

Fig. 7 shows the feature importance with regard to the overall 31 input characteristics. We can see that body height and body weight are the most important factors of echocardiographic LVH with a coefficient magnitude greater than 4 in our SVM model. The other significant predictors of LVH with greater coefficient magnitude include age, heart rate, PR interval, uncorrected QT interval, QRS axis in Lead II, R amplitudes in Lead I, Lead V3, V4, and S amplitudes in Lead V3, V6.

### C. Discussion

A few studies have utilized machine learning or deep learning techniques for ECG characteristics training to predict the LVH presence [1]–[3], [53]–[55]. However, the disadvantages are that most of the studies include a small sample size of participants [53]–[55], or the output is aimed merely for HCM [2], [3] but not for all kinds of LVH phenotypes in the general population. To our knowledge, the Multi-Ethnic Study of Atherosclerosis (MESA) study might be the only one research in screening for the presence of any unspecific LVH based on the definition of cardiac magnetic resonance imaging in a large sample size of middle and old aged general population [1]. As compared with MESA, our study has superior results as both the ECG characteristics and simple biological features are trained by the SVM. On the basis of feature importance analysis of the proposed SVM model, it is obvious that body height and body weight are the strongest predictors of LVH among young adults. We also notice that an addition of systolic and diastolic blood pressures on the currently used SVM model can improve merely a little or is similar in the detection of LVH. It is reasonable that elevated levels of blood pressure are highly correlated with greater body mass index among young adults [56] and the effect time on cardiac remodeling is relatively short. Therefore, blood pressure may not play a critical role on the development of LVH in young adults.

## V. Conclusion

This study develops a clinically effective ECG-based system with age and simple anthropometric data through the SVM machine learning technique in screening for unspecific LVH among young adults, which improves much in the sum of sensitivity and specificity as compared with the traditional ECG criteria for LVH or using the ECG parameters alone for the machine learning. The sensitivity of our proposed method achieves up to 92.6%. In addition, since the test performances regarding accuracy, specificity, sensitivity, precision, F_1_ score, and the AUCs of the ROC and PR curves for the female samples are not optimal by adopting the SVM model trained by male samples, future studies should be done to clarify the validity of our system operated specifically for young females.
